# Supplementary material for: Transmission mode of watermelon silver mottle virus by Thrips palmi
Source: PLoS One. 2021 Mar 3;16(3):e0247500. doi: 10.1371/journal.pone.0247500 (PMC7928467; doi:10.1371/journal.pone.0247500)
Supplement: S1 Table — (PDF) [file pone.0247500.s001.pdf]

S1 Table . Transmission mode and transmission rate of watermelon silver mottle virus by *Thrips palmi*

Transmission mode	Virus-acquiring instar	Virus-inoculating instar	Transmission rate[a]			*P* value[b]
			Trial 1	Trial 2	Trial 3	
Non-persistent transmission						
	First-instar larva	First-instar larva	0/10	0/10	0/10	1.0
	Second-instar larva	Second-instar larva	0/10	0/10	0/10	1.0
	Adult	Adult	0/10	0/10	0/10	1.0
Semi-persistent transmission						
	First-instar larva	First-instar larva	0/10	0/10	1/10	0.29
	Second-instar larva	Second-instar larva	1/10	1/10	0/10	0.37
	Adult	Adult	0/10	0/10	0/10	1.0
Persistent transmission						
	First-instar larva	Adult	8/10	7/10	8/10	0.65
	First-instar larva	Second-instar larva	0/10	1/10	0/10	0.29
	Second-instar larva	Adult	0/10	0/10	1/10	0.29
	Adult	Adult	1/10	1/10	0/10	0.37

[a] The transmission rate was calculated as the ratio of test plants positive for WSMoV examined by RT-PCR assays to total test plants.

[b] Transmission rates among trials were compared using Chi-squared test.
