# Supplementary material for: Transmission mode of watermelon silver mottle virus by Thrips palmi
Source: PLoS One. 2021 Mar 3;16(3):e0247500. doi: 10.1371/journal.pone.0247500 (PMC7928467; doi:10.1371/journal.pone.0247500)
Supplement: S1 Raw images — (PDF) [file pone.0247500.s002.pdf]

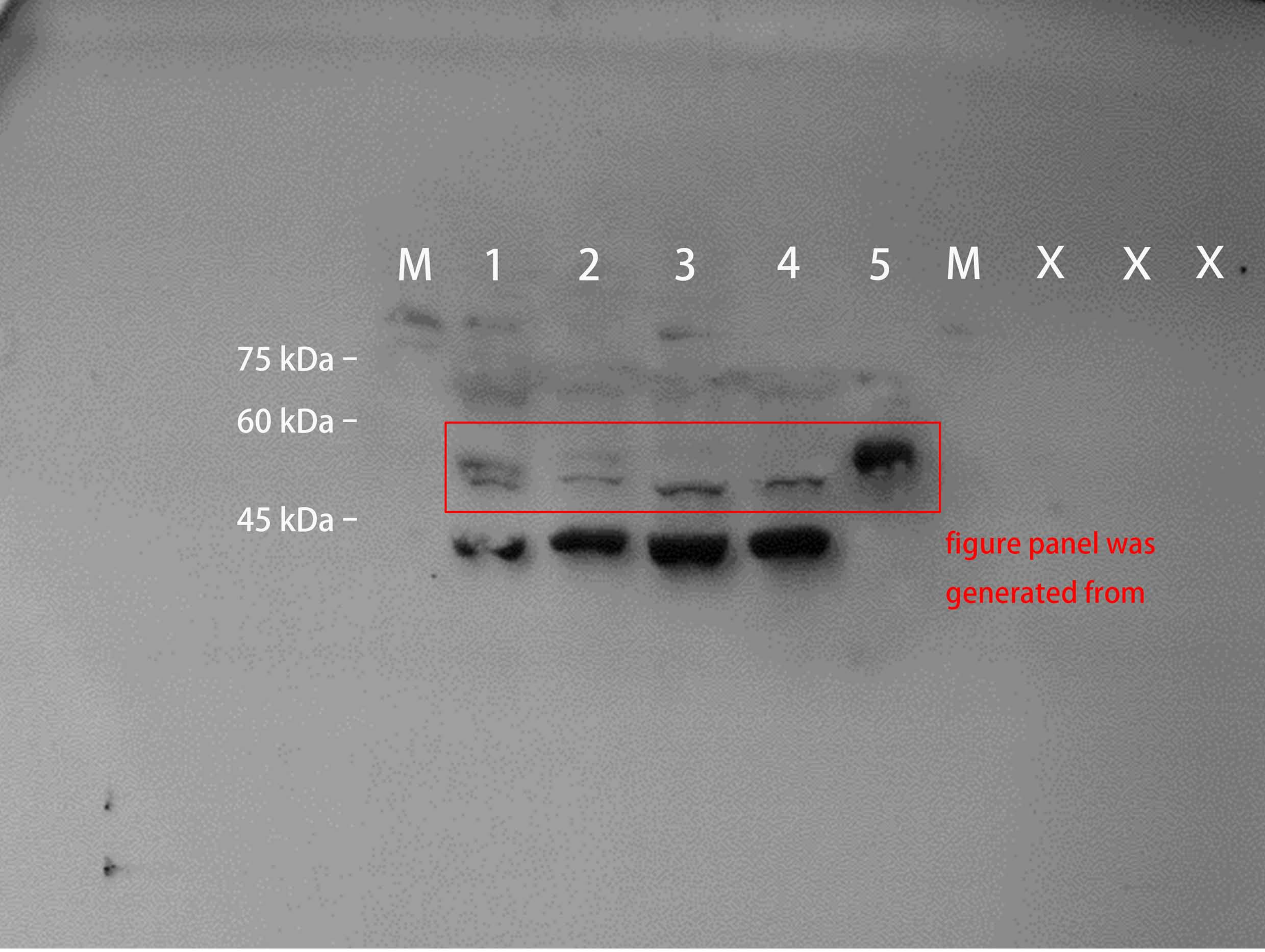

M, Marker

1, 20 viruliferous females

2, 20 viruliferous males

3, 20 non-viruliferous females

4, 20 non-viruliferous males

5, WSMoV-infected watermelon leaf

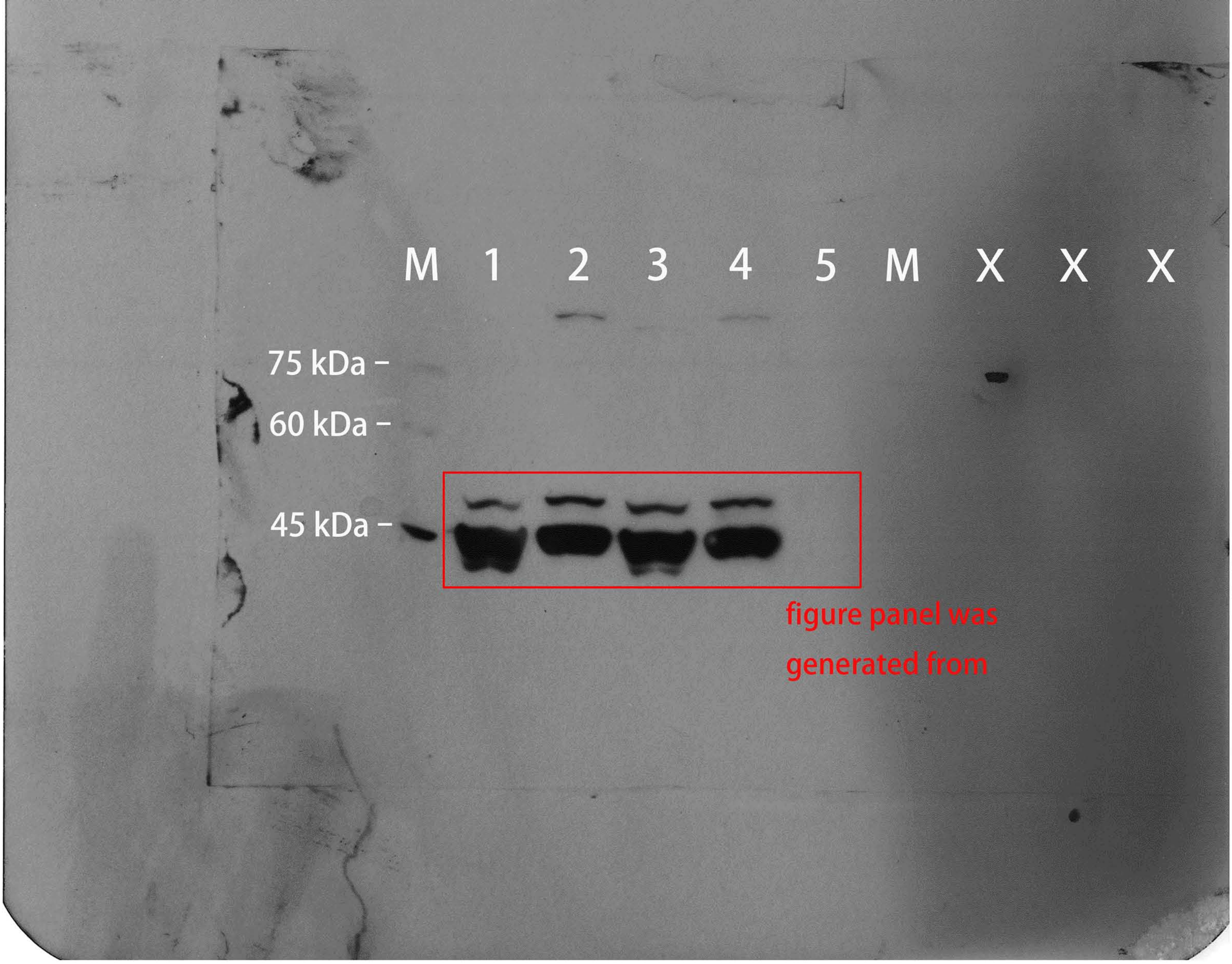

M, Marker

1, 20 viruliferous females

2, 20 viruliferous males

3, 20 non-viruliferous females

4, 20 non-viruliferous males

5, WSMoV-infected watermelon leaf
