# Supplementary material for: Transmission mode of watermelon silver mottle virus by Thrips palmi
Source: PLoS One. 2021 Mar 3;16(3):e0247500. doi: 10.1371/journal.pone.0247500 (PMC7928467; doi:10.1371/journal.pone.0247500)
Supplement: Attachment — Submitted filename: Response to Reviewers.docx [file pone.0247500.s003.docx]

**Response to Reviewers**

Reviewer #1: The manuscript entitled “Transmission mode of watermelon silver mottle virus by Thrips palmi” improves our understanding on the transmission mode of a Tospovirus of global importance. It is well written and it provides relevant information on the ecology of the disease triangle (watermelon- WSMoV-Thrips palmi).

However, I have two major concerns. The first one relates to data analysis. There is a lack of information on the statistical methods used. Only when looking at the results, authors report that they performed logistic regression (253-255). This should be further explained in the materials and methods section, describing the modelling approach, factors taken into consideration, variables and the statistical package used. Also, Table 1 would benefit from reporting sample sizes (I guess from the methods that authors used n=30 per assay, but it is useful to see it already in the table, especially if some replicates were lost).

**Response**: We consulted a statistician, and the data were analyzed again. The information of statistical analyses was explained in the materials and methods section. Sample sizes were added in Table 1 as suggested.

My second major point relates to the status of T. palmi as the only vector of the virus. References 5) and 6) state that this species transmits WSMoV, but, to my knowledge, there are no studies looking at the range of potential vectors that could transmit the virus. In this sense, the argument in lines 64-66 may be misleading. In these sentences, it seems like F. occidentalis is not a potential vector of WSMoV, while this may well be, giving the ability of this thrips species to transmit a wide range of tospoviruses. Additionally (Chiemsombat et al. Arch Virol (2008) 153:571–577 DOI: 10.1007/s00705-007-0024-3) suggested that another thrips species (Scritothrips dorsalis) could potentially vector the virus. I would encourage authors to further explore this aspect in their text.

**Response**: There are no published researches studying the range of potential vectors of WSMoV, so we have rewritten the sentences. Also, there are no studies investigating vector competence of *F. occidentalis* to transmit WSMoV. Chiemsombat et al. (2008) only mentioned that “the ability of these two thrips species to transmit different tospoviruses in Thailand needs further investigation,” but no experiments were performed; therefore, we decide not to cite the paper.

In lines 335-336 authors suggest that there is a disagreement between their findings and those of Chen et al. 2006. I would recommend to expand the discussion on mechanical transmission of WSMoV, for example by addressing the following question: What are the different methodologies and findings between the current and previously published work on transmission mechanisms (Chen et al. 2006)?

**Response**: Chen et al. (2006) proposed that *T. palmi* transmits WSMoV both non-persistently and persistently; however, it is not in line with our understanding of virus transmission mode. We evaluated the methods of their experiments and found they were okay. Therefore, we decided to conduct a series of experiments to examine whether *T. palmi* transmits WSMoV non-persistently, semi-persistently, or persistently. Finally, our results clarify that *T. palmi* transmits WSMoV in a persistent-propagative mode, and not in a non-persistent or semi-persistent manner.

Few minor points:

-line 98: 150 mesh: please, provide the number of threads/cm2 or the area of the opening.

**Response**: The information (pore size 160 μm) was added as suggested.

-line 184: Potential viruliferous or “potentially viruliferous”?

**Response**: It was corrected.

-line 227: In the era of Open Access, are authors going to deposit their sequences in the public database of ncbi?

**Response**: The sequences of partial *NSm* and *NSs* genes were 100% identical to WSMoV isolate DD6 the M and S segments in GenBank (accession numbers DQ157768 and AY864852, respectively). The information was added in the text.

-line 332: Additionally, Frankliniella fusca is a major vector of TSWV in peanuts, for example.

**Response**: Thank you for providing the reference. We have added the information and cited the paper (Sakimura, 1963. Phytopathology).

Reviewer #2: The authors aimed to empirically determine the transmission mode of WSMoV, an orthotospovirus, by Thrips palmi, the reported thrips vector of this plant virus. The type member of the genus orthotospovirus (tomato spotted wilt virus) is transmitted by other thrips vectors in a persistent - propagative manner, meaning that the virus replicates in the vector and disseminates from the midgut to the salivary glands prior to inoculation of plants. As such, the authors performed various transmission experiments with various parameters to distinguish some transmission modes, along with confocal laser scanning microscopy of immunofluorescent detection of WSMoV nucleocapsid (N) protein (structural protein) to document the presence of the viral protein in adult thrips tissues known to be associated with orthotospovirus infection.

Major Concerns:

1. The authors rationalize that endpoint RT-PCR detection of complementary RNA of the NSs or NSm genes is evidence of replication of WSMoV. While it is correct that amplification of the viral complement (vc) of NSs (S genome segment) or NSm (M genome segment) is suggestive of replication (these viral proteins are translated from subgenomic virus sense mRNA), it is not conclusive of replication because it has been reported in foundational studies that the vc of M and/or S RNA segments of TSWV and other orthotospoviruses (e.g., INSV) can be encapsidated in the virion [VIROLOGY 188, 732-741 (1992) (Law et al); Journal of General Virology (1992), 73, 687-693 (Kormelink et al)]. As such, to be rigorous, it is strongly recommended that the authors perform additional experiments to definitively show replication of WSMoV in Thrips palmi - such as evidence of NSs or NSm protein in the thrips vector using western blot analysis or immunolocalization in tissues with antibodies to these nonstructural viral proteins, because to produce these nonstructural proteins, the S and M RNAs must be replicated into full length vc strands followed by mRNA synthesis. The authors chose to use antibodies to the N protein (structural protein) in their confocal microscopy observation of virus in thrips tissues, so they clearly have the skills to dissect and fix thrips tissues and to perform and analyze confocal microscope images. It is recommended they perform these tissue dissections and immunofluorescence with antibodies to NSs or NSm. Also, use of real-time quantitative RT-PCR of NSs and NSm to estimate virus accumulation (increase) over time after removal of viral inoculum may also support their conclusions.

**Response**: We agree with the reviewer’s comment that the detection of complementary RNA of the NSs and NSm genes is not conclusive of virus replication. Therefore, we performed western blot analysis of NSs protein in viruliferous thrips to demonstrate the replication of WSMoV in *Thrips palmi* (Fig. 4).

2. The introduction is missing key elements to help the reader understand the logic and rationale of the objective/subobjectives of the study. There is no mention of the genome organization of WSMoV or other orthotospoviruses, which would help readers understand the authors' approach. WSMoV genome is deposited in NCBI Genbank. There is no clear description of possible transmission modes - nonpersistent, semipersistent, persistent non-propagative, persistent-propagative - that set the stage for the acquisition-inoculation experiments. Along these lines, a description of factors that vector biologists use to distinguish transmission modes (i.e., time of virus exposure (AAP), time to virus delivery (IAP), latent period, frequency of inoculation, accumulation of virions and/or nonstructural proteins). It is unclear to the reader how the authors differentiate or identify the different modes of transmission, and therefore the manuscript would benefit from a clear description in the introduction. And, on lines 87 - 90, there is mention of phylogenetic analysis of N aa sequences between WSMoV and TSWV, but there is no clear logic as to why this is mentioned or relevant to the study.

**Response**: The background information of transmission mode of insect transmitted plant viruses was added in the introduction section. The phylogenetic analysis of amino acid sequences of WSMoV and TSWV N proteins demonstrates that WSMoV is distantly related to TSWV. Our objective was to examine the transmission mode of a virus that is distantly related to well-studied prototype, TSWV. We have rewritten the sentences. To avoid an overly long introduction, we did not add the description of WSMoV genome organization because the genome organization of WSMoV is the same as that of other orthotospoviruses.

3. Lines 78 - 86. The assertion that published studies make assumptions about orthotospovirus transmission biology based on TSWV is not fairly supported. In fact, several of the references cited by the authors on line 82 have examined transmission and accumulation of viral protein (nonstructural) of other tospoviruses and different thrips species. It is strongly suggested that this assertion and text be modified to accurately reflect the knowledge of transmission modes of orthotospoviruses and to temper the language as to not offend experts in the field. Along this line, the authors missed a recent study that documented vector competence of another orthotospovirus, soybean vein necrosis virus (SVNV) by soybean thrips (Neohydatothrips variabilis) that carried out the appropriate steps (testing different AAPs and IAPs and confocal microscopy to trace tissue tropism in the vector) to determine transmission - no assumptions were made.

**Response**: We thank the reviewer for the kind reminding. We have no intention to offend experts in this field. The paragraph addresses the fact that these studies, transmission mode of tospoviruses other than TSWV, only examined the possibility of persistent transmission but not the possibility of non-persistent and semi-persistent transmission modes. We did not mention that these studies did not demonstrate the accumulation of viral nonstructural proteins and virus replication. We have rewritten the sentences to make it more clear. We apologize for missing the recent study of SVNV (Han et al. 2019. Front. Microbiol.), and the paper was cited. However, Han et al. (2019) let first-instar larvae to acquire the virus and inoculated soybean leaf disk with adult thrips. They also did not examine the possibility of non-persistent and semi-persistent transmission modes for SVNV.

In addition, it seems odd that members of this author group published unrelated research with this vector (T. palmi) and orthotospovirus species (WSMoV) and appeared to work out the AAP and IAP for their experiments (https://doi.org/10.1371/journal.pone.0102021). They produced WSMoV-infected plants under the presumption that L1s acquire the virus (24 hour AAP) and adults transmit (IAP), so it would appear that in this previous paper, the authors presumed that WSMoV is transmitted in the same manner as a persistent virus like TSWV (virus acquired as young L1s, virus retained after molting and pupation, adults inoculate plants).

**Response**: That's right. We presumed that vector transmission of WSMoV is the same as that of TSWV in our previous published paper. Examined thrips acquired the virus at L1 stage and transmitted the virus after emergence as adults. We thought that the transmission of WSMoV needs to be examined in details, and this was our motivation for this research.

4. Lack of statistical analysis section for handling transmission data. The authors mention 'logistic regression' (line 255) of the transmission efficiency data in Table 1, but how did the authors handle the three independent experiments, were data pooled, what was the statistical model tested, were assumptions met? Please devote a few lines to describing the value of the 'Swallow estimator' and why it was used here.

**Response**: We consulted a statistician, and the data were analyzed again. The information of statistical analyses was explained in the materials and methods section. Swallow estimator was used to estimate the probability that a single thrips transmits the virus when groups of thrips were used for virus inoculation. The information was moved to Statistical analyses section in the materials and methods.

Minor comment: The writing could be improved throughout; there were several awkward clauses and ambiguous language.

**Response**: English is not our native language, so the manuscript has been edited by a native speaker, Ms. Reina L. Tong, in the submitted version. The revised manuscript has been carefully revised by a professional language editing service to improve the grammar and readability.
