# Supplementary material for: Transmission mode of watermelon silver mottle virus by Thrips palmi
Source: PLoS One. 2021 Mar 3;16(3):e0247500. doi: 10.1371/journal.pone.0247500 (PMC7928467; doi:10.1371/journal.pone.0247500)
Supplement: Attachment — Submitted filename: Response to Reviewers.docx [file pone.0247500.s004.docx]

**Response to Reviewers**

Reviewer #1: I would like to acknowledge that authors addressed well my previous comments to the manuscript entitled “Transmission mode of watermelon silver mottle virus by Thrips palmi”. I have only two considerations in relation to the section of statistics and table 1.

Authors use reference number 38 (Chen et al. 2006) to refer to the calculations of the Swallow estimator (line 266) However, based on the list provided, it is likely that the reference that correspond to the definition of this estimator is number 37 (Swallow, 1985). Therefore, I would suggest to correct the numbering of the reference list and to double check that all the references are in agreement with the statements in the text.

**Response:** The citations in the manuscript are carefully double-checked.

In addition, in table 1, there is one cell in the column “Ps” that remains empty. This corresponds to thrips that acquired the virus as first instar larvae and transmitted as adults. I would encourage authors to also provide this value to complete the information. Also, the formula of Ps could be added to the figure footnotes so that readers can figure out the meaning of this parameter.

**Response:** Because one thrips per test plant was used for virus inoculation, there is no Swallow estimator for the cell in Table 1. The formula of Ps is added to the footnotes.

Reviewer #2: The authors responded adequately to my comments in the narrative and in performing the additional experiments to provide more supportive evidence for persistent/propagative (viral replication) transmission of WSMoV by T. palmi. This study provides a thorough examination of this orthotospovirus/thrips vector transmission mode. The revised manuscript is more clearly written and presented logically (the rationale for the study is clearly stated in this version), and it contains sufficient background and description of methodology to evaluate the significance of the study

Below are a few minor comments to address in the final revision:

Figures and figure legends.

Figures should stand alone with the use of legends, meaning, the reader should not have to revisit the paper methodologies to fully evaluate and comprehend the content of the figure.

Thus figure legends should include pertinent information:

In both figure 1 and 2 legends, there is no mention of the viral protein detected by the antibody: N or Nsm or Nss? The reader should know this from the legends.

**Response:** Nucleocapsid protein was detected by the antibody. Information of the antibody is added to Figures 1 and 2 legends.

Also, in Figure 2, are the five panels in each column showing a different independent sample, eg. A, E, I, M and Q are internal organs of five different individuals from how many of the repeated experiments? Or a different part of the same tissue of one individual in each column?

Since the authors labeled each panel with letters, they must be meaningful so they should be referred to in the figure legend. Also, in the Results section where Figure 2 is reported, the reader should be told how many samples were tested and if what's depicted in Figure 2 is a representative sample.

**Response:** Yes, they are confocal images of organs from five different individuals. Figure 2 is modified, and the figure legend is edited. In the Results section, the information is already described in the manuscript (lines 332-4).

Statistical methods section.

Statistics - data was pooled from three biological repetitions of the transmission experiment (Table 1) because authors found that "there were no significant differences of variance among them." (line 292-293). Please include the name of the statistical test performed and provide the test statistic used to assess this. Also, it is increasingly more common to provide supplementary/supportive materials with manuscripts for publication. As such, it is recommended that an excel file or word table be provided that shows % transmission data for each of the three biological repetitions to show repeatability/robustness of findings and thoroughness of the study.

**Response:** The statistical test performed to examine variance among 3 replicates is added (lines 304-5 and 316-8). We also add a supplementary table as suggested.
